# Mast Cells Drive Systemic Inflammation and Compromised Bone Repair After Trauma

**DOI:** 10.3389/fimmu.2022.883707

**Published:** 2022-04-26

**Authors:** Deniz Ragipoglu, Jasmin Bülow, Kristin Hauff, Martin Voss, Melanie Haffner-Luntzer, Anne Dudeck, Anita Ignatius, Verena Fischer

**Affiliations:** ^1^ Institute of Orthopedic Research and Biomechanics, Trauma Research Center Ulm (ZTF), Ulm University Medical Center, Ulm, Germany; ^2^ Medical Faculty, Institute for Molecular and Clinical Immunology, Otto-von-Guericke University Magdeburg, Magdeburg, Germany

**Keywords:** mast cells, posttraumatic inflammation, fracture healing, trauma, interleukin-6

## Abstract

There is evidence that mast cells contribute to inflammation induced by hemorrhagic shock, severe tissue injury or sepsis. Mast cells are highly responsive to alarm signals generated after trauma, and release many inflammatory mediators including interleukin-6, a key mediator of posttraumatic inflammation. An overwhelming posttraumatic inflammation causes compromised bone healing; however, the underlying cellular and molecular mechanisms are poorly understood. Recently, we found that mast cells trigger local and systemic inflammation after isolated fracture leading to uneventful bone repair. Here, we investigated whether mast cells critically contribute to trauma-induced compromised bone healing. Male Mcpt5-Cre^+^ R-DTA mice, which lack connective tissue type mast cells, and their mast cell-competent Cre^−^ littermates underwent a femur fracture with/without thoracic trauma. Posttraumatic systemic and local inflammation and bone repair were assessed 3 h and 21 d post injury. Both, the systemic and pulmonary inflammation was significantly increased in mast cell-competent mice upon combined trauma compared to isolated fracture. In mast cell-deficient mice, the increase of inflammatory mediators in the circulation induced by the severe trauma was abolished. In the bronchoalveolar lavage fluid, the trauma-induced increase of inflammatory cytokines was not reduced, but the neutrophil invasion into the lungs was significantly diminished in the absence of mast cells. Locally in the fracture hematoma, mast cell-competent mice displayed reduced inflammatory mediator concentrations after combined trauma compared to isolated fracture, which was abolished in mast cell-deficient mice. Notably, while combined trauma resulted in compromised bone repair in mast cell-competent mice, indicated by significantly reduced bone and increased cartilage fracture callus contents, this was abolished in Mcpt5-Cre^+^ R-DTA mice. Therefore, mast cells contribute to trauma-induced compromised bone repair and could be a potential target for new treatment options to improve fracture healing in multiply injured patients.

## Introduction

One of the most common forms of injuries in multi-trauma patients are extremity fractures. Depending on trauma severity, the risk for fracture malunion increases up to 50% (1, 2). Tissue trauma induces a systemic posttraumatic immune response triggered by damage-associated molecular patterns (DAMPs) being released from injured tissues (3). In multiply injured patients, the blunt chest trauma is clinically highly relevant, as it represents one of the most critical injures and is regarded as an important trigger of the systemic inflammatory response (3–5). Furthermore, 50% of patients with blunt chest trauma are additionally affected by fractures (6). The systemic hyper-inflammation after trauma is characterized by immune cell activation and a storm of pro-inflammatory mediators (3), which might negatively interfere with the inflammation and regenerative processes locally at the fracture site (7–10). In this line, it was demonstrated that the immune cell composition in the fracture hematoma was altered in a murine model of combined fracture and thoracic trauma, with a significantly higher number of neutrophils and a decreased number of macrophages (11, 12). In pigs, the levels of inflammatory mediators including interleukin-6 (IL-6), IL-8, IL-10 and the alarmin high-mobility-group-protein B-1 were altered in the fracture hematoma after an additional chest or polytrauma (13, 14). In humans, impaired bone healing was associated with altered leucocyte kinetics in multi-trauma patients (15). Furthermore, the blockade of posttraumatic inflammation appears to improve fracture healing after severe trauma. For example, it was demonstrated that IL-6, a key inflammatory cytokine in the posttraumatic immune response (16), may play a critical role in trauma-induced compromised fracture healing, because the selective blockade of IL-6 trans-signaling attenuated the deleterious effects of an additional blunt chest trauma on bone repair in mice (17). Similarly, the blockade of the complement anaphylatoxin receptor C5aR1 reduced the systemic immune response and ameliorated trauma-induced impaired fracture healing (18). In summary, these studies suggest that disturbed inflammatory processes and immune cell functions play an essential role in compromised bone healing after trauma; however, the underlying mechanisms remain poorly understood.

Mast cells store and *de novo* synthesize numerous mediators, including IL-6, tumor necrosis factor (TNF), histamine, heparin, enzymes, and growth factors. Acting as immunological guards, these cells are mainly located in tissues exposed to the external environment, including skin, intestines, and lungs but also in the bone marrow (19, 20). Because mast cells are highly responsive to early alarm signals that are generated after trauma including DAMPs and complement anaphylatoxins (19), they may be one of the critical drivers in posttraumatic inflammation and trauma-induced compromised fracture healing. Supporting this, some authors demonstrated that mast cells contribute to the inflammatory response induced by hemorrhagic shock, severe tissue injury, or ischemia-reperfusion injury (21–23). For example, the inhibition of mast cell degranulation with the mast cell-stabilizer cromolyn ameliorated severe consequences of ischemia-reperfusion injury in rodents (24). Moreover, the inflammatory response and multiple organ injury were attenuated after hemorrhagic shock in mast cell-deficient mice compared to wildtype mice (21), and after mast cell stabilization using ketotifen and cromolyn in rats (25).

Mast cells appear to also play an important role in fracture healing (26–28). Our group demonstrated that bone resident mast cells trigger local and systemic inflammatory processes after an isolated fracture, a model for undisturbed bone repair. In mast cell-deficient Mcpt5-Cre R-DTA mice, which lack connective tissue mast cells, the levels of proinflammatory cytokines, including IL-6, IL-1β, and interferon-γ, and the number of neutrophils and macrophages were significantly reduced in the fracture hematoma. In addition, systemic levels of pro-inflammatory cytokines were also reduced in the absence of mast cells (26). Moreover, mast cells might contribute to bone formation and callus remodeling during bone repair (27, 28), because they were shown, for example, to stimulate osteoclast formation and activity during fracture callus resorption (26). Recently, we additionally revealed that mast cells were responsible for the increased fracture-induced systemic and local inflammatory response and for compromised healing in osteoporotic bone (29, 30). These data suggest that mast cells regulate inflammation and repair during bone fracture healing. However, their role in compromised fracture healing after severe trauma has, to date, not been elucidated.

The present study aimed to investigate, whether mast cells critically contribute to trauma-induced compromised bone repair. To this end, we exposed mast cell-deficient Mcpt5-Cre R-DTA mice to an isolated femur fracture, which heals uneventfully, or to combined fracture and thoracic trauma, inducing more severe inflammation and compromised fracture healing. We demonstrated, that mast cell-deficient mice displayed a balanced systemic and local inflammatory response and were protected from compromised bone healing after severe trauma. Therefore, this study implies that mast cells could be a target for new therapeutic strategies to improve bone repair in multiply injured patients.

## Materials and Methods

### Study Design

The experiments were performed in male Mcpt5-Cre R-DTA mice (C57BL/6J background). These mice express diphtheria toxin (DT) under the control of the Mcpt5 promoter, which is specific for connective tissue type mast cells and drives Cre-specific mast cell ablation in these mice (mast cell-deficient). Male Mcpt5-Cre^−^ R-DTA littermates were used as mast cell-competent controls. Mice were bred as described previously (31, 32). The animals were housed in groups of two to five mice under standard rodent conditions. When aged 12 weeks, the mice were randomly assigned to the different treatment groups either subjected to isolated femur fracture (Fx) or a combined fracture and thoracic trauma (Fx + TxT). Mice were euthanized 3 h, 6 h and 21 d after injury (n = 3–8 per group and time point) using isoflurane overdose. Blood, bronchoalveolar lavage (BAL) fluids and tissues (fracture hematoma, femur, lung, liver) were harvested for multiplex analysis (blood, BAL), tissue histology (lung, femur), gene expression analysis (liver), and µCT and biomechanical testing (femur). Samples were allocated in a blinded manner to the different analyses, which were performed only on a subset of tissue specimens due to technical reasons. All animal experiments were performed in compliance with international regulations for the care and use of laboratory animals (ARRIVE guidelines and EU Directive 2010/63/EU for animal experiments) with the approval of the Local Ethical Committee (no. 1386, Regierungspraesidium Tuebingen, Germany).

### Femur Osteotomy and Thoracic Trauma

When aged 12 weeks, all mice were subjected to a standardized unilateral femur osteotomy as described previously (33). Briefly, under general anesthesia with 2% isoflurane, an osteotomy gap (gap size: 0.44 mm) was created at the mid-shaft of the femur using a 0.44 mm Gigli wire saw (RISystem, Davos, Switzerland) that was stabilized with an external fixator (axial stiffness 3 N/mm, RISystem). The external fixator prevents torsional stability, which results in defined and standardized biomechanical conditions that greatly influence the fracture healing process, and does not impede with biological reactions at the fracture site (33, 34). Prior to the surgery the mice received one subcutaneous injection of the antibiotic clindamycin-2-dihydrogenphosphate (45 mg/kg, Ratiopharm, Ulm, Germany). Half of the mast cell-competent and mast cell-deficient mice received an additional thoracic trauma immediately after the fracture while the mice were still under general anesthesia as previously described (11, 17). Briefly, using a blast-wave generator, a single blast wave was applied to the middle of the thorax, which produces a standardized bilateral, isolated lung contusion. For pain treatment, all mice received tramadol hydrochloride (25 mg/l) in the drinking water starting 1 d pre-surgery until 3 d post-surgery.

### Multiplex Cytokine Analysis and ELISA

To assess the systemic and local inflammation, serum, BAL, and hematoma were obtained 3 h post-fracture. The lungs were flushed with ice-cold phosphate-buffered saline, and BAL fluids were centrifuged at 300 × *g* for 15 min (35). The fracture hematoma was harvested and lysed as previously described (17). A customized mouse Multiplex Cytokine Kit (ProcartaPlex, eBioscience, Frankfurt, Germany) was used to determine blood serum, BAL, and hematoma concentrations of the following inflammatory cytokines and chemokines using the Bio-Plex 200 System (Bio-Rad Laboratories, Hercules, CA, USA): IL-6, IL-5, C-X-C motif chemokine-10 (CXCL-10), monocyte chemoattractant protein-1 (MCP-1), MCP-3, eotaxin, CXCL-1, IL-10, IL-9, and IL-3. Instrument validation was performed monthly using the Bio-Plex Validation Kit (Bio-Rad Laboratories) and calibration was performed prior to sample measurement using the Bio-Plex Calibration Kit (Bio-Rad Laboratories) according to the manufacturers protocol, respectively. Total protein concentration of the fracture hematoma was determined with the Pierce™ BCA Protein Assay Kit and the cytokine concentrations were normalized to total protein.

### Liver Homogenates and Real-Time PCR

Liver lobes were harvested 3 h after surgery, immediately frozen in liquid nitrogen, and stored at −80°C until homogenates were prepared. A small piece was taken from each frozen liver sample, and homogenized in Trizol, using a homogenisator (MICCRA D-9, MICCRA GmbH, Müllheim, Germany), and incubated for 5 min at room temperature (RT). Subsequently, 200 µl chloroform were added and vigorously vortexed before centrifugation at 12,000 × *g* for 30 min. Following centrifugation, the supernatants were collected, and the RNA was isolated using the PureLink^®^ Mini Kit (Thermo Fisher Scientific, Waltham, MA, USA) according to the manufacturer’s recommendations. The isolated RNA was diluted in RNase-free water after DNase digestion. Further processing and qPCR analysis were performed as previously described (36, 37). Glyceraldehyde 3-phosphate dehydrogenase (*Gapdh*) served as a housekeeping gene (F: 5′-ACC CAG AAG ACT GTG GAT GG-3′, R: 5′-GGA TGC AGG GAT GAT GTT CT-3′). The expression of chemokines and acute-phase proteins was determined using specific primers for *Cxcl-1 (*F: 5′-TCT CCG TTA CTT GGG GAC AC-3′, R: 5′-CCA CAC TCA AGA ATG GTC GC-3′), and C-reactive protein (*Crp)* (F: 5′-ATC CCA GCA GCA TCC ATA GC-3′, R: 5′-AAC ATG TCT TCA TGA CCA AAA GTC C-3′). Relative gene expression was calculated by normalizing to the house keeping gene *Gapdh* using the delta-delta CT method.

### Lung Histomorphometry and Immunohistochemistry

Lungs harvested after 3 h and 6 h were fixed in 4% paraformaldehyde for 48 h and embedded in paraffin. For morphological analysis of lung injury, paraffin-embedded lung sections harvested 3 h after surgery were stained with hematoxylin and eosin. Neutrophil granulocytes and mucosal mast cells were stained using Ly6G antibody (1:200, BioLegend, CA, USA) and Mcpt-1 antibody (1:100, Invitrogen, MA, USA), 3 h and 6 h after surgery, respectively. To this end, lung sections were deparaffinized in xylene, rehydrated in descending ethanol gradient, and boiled in sodium citrate buffer for antigen retrieval. Sections were blocked with 5% goat serum for 1h at RT and incubated with the primary antibodies. Species-specific IgG was used as negative control for Ly6G staining and rabbit serum for Mcpt-1 staining. As secondary antibody, goat anti-rat IgG-biotin (1:200 and 1:100 (Life Technologies, Carlsbad, CA, USA), respectively, for Mcpt-1 and Ly6G staining) was used and incubated at RT for 1 h (Mcpt-1) or 30 min (Ly6G). For signal detection, the Vectastain Elite ABC kit and Vector NovaRed substrate (both Vector laboratories Inc., Burlingame, USA) were used according to the manufacturer’s instructions. Sections were counterstained with hematoxylin and analyzed by light microscopy (Leica DMI600B, Leica, Wetzlar, Germany).

### Biomechanical Testing

To evaluate the mechanical competence of healed femurs explanted on d 21, a non-destructive three-point bending test was performed as described previously (33). In brief, the external fixator was removed and femurs were fixed with aluminum cylinders into a material testing machine (Zwick Roell, Ulm, Germany). An axial load with a maximum of 2 N was applied to the top of the fracture callus at the midshaft of the femur and the load and deflection were recorded. The bending stiffness of the fractured bone was calculated from the slope of the load-deflection curve.

### Micro-Computed Tomography (µCT) Analysis

Following biomechanical testing, fractured femurs were fixed in 4% paraformaldehyde for 48 h. Femurs were scanned using a µCT device (Skyscan 1172; Bruker, Kontrich, Belgium) to evaluate the structural properties of the fracture calli at an isotropic voxel resolution of 8 µm using a voltage of 50 kV and 200 mA. Two phantoms with a defined hydroxyapatite (HA) content were used for calibration and the assessment of bone mineral density (250 and 750 mgHA/cm^3^). The volume of interest comprised the entire periosteal callus between the two inner pin-holes. A global threshold for mineralized tissue was set at 642 mgHA/cm^3^ and data were analyzed according to Bouxsein et al. (38).

### Fracture Calli Histomorphometry and Immunohistochemistry

Histological analysis was performed on fracture calli on d 21 post-fracture. Following µCT analysis, fractured bones were decalcified using 20% ethylenediamine tetraacetic acid (pH 7.2–7.4) and embedded in paraffin. Sections of 7 µm were stained with Safranin O (Sigma Aldrich, St. Louis, MO, USA) and the relative amounts of bone, cartilage and soft tissue were evaluated by image analysis (Software MMAF 1.4.0 MetaMorph, Leica) using a light microscope (Leica DMI600B, Leica). The fracture gap was defined as a region of interest. Osteoblasts were counted in Toluidine blue-stained sections and osteoclasts in sections stained for tartrate-resistant acid phosphatase in a rectangular area (650 × 450 μm) in the middle of the fracture callus.

For immunofluorescence staining, 4 µm thick sections of the fracture calli on d 21 post-fracture were prepared and Avidin Texas Red conjugated antibody (Thermo Fisher Scientific, MA, USA) was used to detect connective tissue type mast cells. Bone sections were deparaffinized, rehydrated, and antigen retrieval was performed using sodium citrate buffer boiling, followed by blocking with 5% bovine serum albumin and incubation with the Avidin Texas Red antibody (1:300) for 1 h at RT. Mast cell-deficient mice were used as negative controls. For signal detection, a spectrum with excitation and emission maxima of ~595/615 nm was used. Hoechst (1:2000) was used for counterstaining and sections were analyzed by light microscopy (Leica DMI600B, Leica). Mast cells were counted in the entire periosteal callus and the bone marrow between the two inner pinholes.

### Statistical Analysis

In all figures, data are presented as box-and-whisker plots (with the median and interquartile range) from maximum to minimum, showing all data points. Data in tables are presented as mean ± standard deviation. Data were tested for normal distribution using Shapiro-Wilk normality test. Comparisons between two groups were either performed by two-tailed Student’s t-test in the case of normal distribution or Mann-Whitney U test if data were not normally distributed. Comparisons between more than two groups were either performed by one way analysis of variance (ANOVA) and Fishers LSD *post hoc* test in the case of normal distribution or Kruskal-Wallis and Dunn’s *post hoc* test if data were not normally distributed. Statistical analysis was performed using GraphPad Prism 9.0 software (GraphPad Software, La Jolla, CA, USA). The level of significance was set at p<0.05. The sample numbers are indicated in the figures.

## Results

No animals were lost during anaesthesia, however, 3 animals died after thoracic trauma. Furthermore, we excluded 4 animals due to post-surgery complications (sample handling, fracture gap size), which resulted in a total complication rate of 10%. All animals tolerated the external fixator well and showed normal and physiological limb loading few days after fracture.

### Mast Cell-Deficiency Attenuates Trauma-Induced Systemic Inflammation

**Figure 1:**
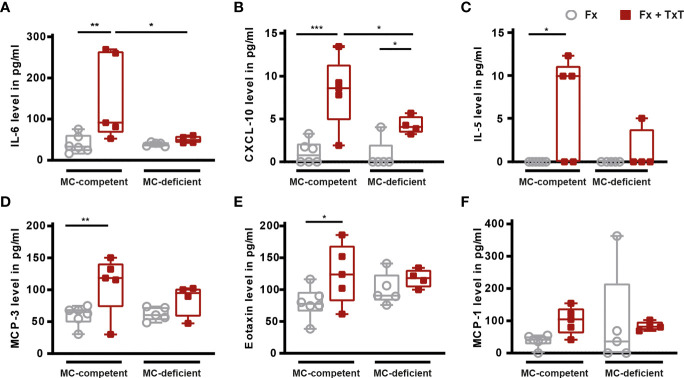
Inflammatory mediator concentrations in serum of mast cell (MC)-competent and MC-deficient mice 3 h after isolated fracture (Fx) and combined fracture and thoracic trauma (Fx + TxT). Serum **(A)** interleukin-6 (IL-6), **(B)** C-X-C motif chemokine ligand 10 (CXCL-10), **(C)** IL-5, **(D)** monocyte chemoattractant protein-3 (MCP-3), **(E)** eotaxin, and **(F)** MCP-1 levels. Gray boxes represent Fx mice, red boxes represent Fx + TxT mice. Data are shown as box-and-whisker plots (with median and interquartile range) from maximum to minimum, showing all data points. *p<0.05; **p<0.01; ***p<0.001. The p values were determined using one-way ANOVA with *post hoc* Fisher’s LSD in the case of normal distribution or with Kruskal-Wallis with *post hoc* Dunn’s test if data were not normally distributed.

To investigate the relevance of mast cells in compromised bone repair after severe trauma, we subjected mast cell-competent and -deficient mice to an isolated femur fracture or combined fracture and thoracic trauma. Confirming our previous studies, in mast cell-competent control mice, the additional thoracic trauma led to an increased inflammatory response 3 h after fracture, as indicated by significantly increased serum IL-6, IL-5, CXCL-10, MCP-3, and eotaxin levels compared to mice with an isolated fracture (**Figures 1A** **–E**). There were no significant differences in MCP-1 levels between the groups detectable (**Figure 1F**). In **Table 1** all other inflammatory mediators are displayed that were detectable in the serum. Notably, mast cell-deficient mice did not display increased serum concentrations of inflammatory cytokines after combined trauma, with the exception of CXCL-10, the levels of which were significantly increased compared to isolated fracture (**Figures 1A** **–E**). However, serum IL-6 and CXCL-10 levels were significantly reduced compared to mast cell-competent mice after additional thoracic trauma (**Figures 1A, B**).

**Table 1:** Inflammatory mediator levels in serum and BAL fluids.

	MC-Competent		MC-Deficient	
	Fx	Fx + TxT	Fx	Fx + TxT
Serum				
IL-17a	3.9 ± 4.0	7.4 ± 14.0	0.6 ± 0.8	0.7 ± 0.8
CXCL-1	266.5 ± 182.3	420.6 ± 207.7	126.4 ± 124.8	269.1 ± 205.7
BAL				
CXCL-1	3.4 ± 7.2	51.0 ± 83.0	1.8 ± 4.1	40.1 ± 50.1
IL-10	17.7 ± 16.8	17.1 ± 13.5	18.1 ± 11.7	2.2 ± 3.2
IL-13	3.7 ± 2.5	3.9 ± 2.8	5.0 ± 4.1	0.8 ± 1.2*
IL-9	58.7 ± 56.4	38.7 ± 55.3	79.8 ± 80.6	0.0 ± 0.0*

*Significantly different (p<0.05) compared to the Fx group, one-way ANOVA with post hoc Fisher’s LSD in the case of normal distribution or with Kruskal-Wallis with post hoc Dunn’s test if data were not normally distributed. MC, mast cell; Fx, isolated fracture; Fx + TxT, fracture and additional thoracic trauma; IL, Interleukin; CXCL, C-X-C motif chemokine ligand; BAL, bronchoalveolar lavage.

**Figure 2:**
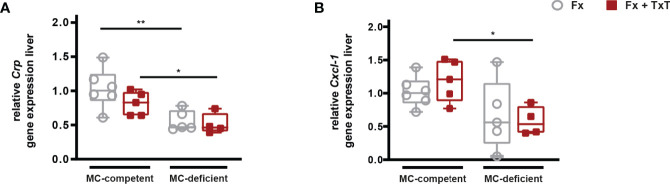
Hepatic acute-phase reaction in mast cell (MC)-competent and MC-deficient mice 3 h after fracture (Fx) and combined fracture and thoracic trauma (Fx + TxT). Relative gene expression of **(A)** C-reactive protein (*Crp*) and **(B)** C-X-C motif chemokine ligand 1 (*Cxcl-1*) in the liver. Relative gene expression was calculated by normalizing to the house keeping gene *Gapdh*. Gray boxes represent Fx mice, red boxes represent Fx + TxT mice. Data are shown as box-and-whisker plots (with median and interquartile range) from maximum to minimum, showing all data points. *p<0.05; **p<0.01. The p values were determined using one-way ANOVA with *post hoc* Fisher’s LSD.

Furthermore, the systemic hepatic acute-phase response was strongly reduced in the absence of mast cells after isolated fracture, as well as after combined fracture with thoracic trauma, as indicated by significantly reduced *Crp* and *Cxcl-1* gene expression compared to the respective mast cell-competent groups (**Figures 2A, B**). In summary, mast cell-deficiency attenuated the early trauma-induced systemic inflammation.

### Mast Cell-Deficiency Only Marginally Influences Trauma-Induced Lung Inflammation

**Figure 3:**
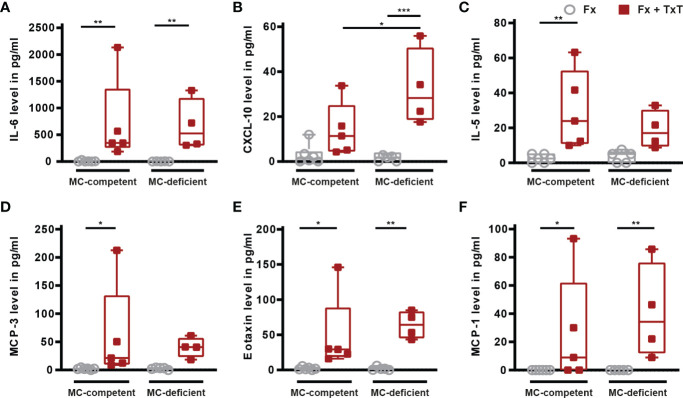
Inflammatory mediator concentrations in bronchoalveolar lavage (BAL) fluid samples of mast cell (MC)-competent and MC-deficient mice 3 h after isolated fracture (Fx) and combined fracture and thoracic trauma (Fx + TxT). BAL fluids **(A)** interleukin-6 (IL-6), **(B)** C-X-C motif chemokine ligand 10 (CXCL-10), **(C)** IL-5, **(D)** monocyte chemoattractant protein-3 (MCP-3), **(E)** eotaxin, and **(F)** MCP-1 levels. Gray boxes represent Fx mice, red boxes represent Fx + TxT mice. Data are shown as box-and-whisker plots (with median and interquartile range) from maximum to minimum, showing all data points. *p<0.05; **p<0.01; ***p<0.001. The p values were determined using one-way ANOVA with *post hoc* Fisher’s LSD in the case of normal distribution or with Kruskal-Wallis with *post hoc* Dunn’s test if data were not normally distributed.

In the lungs, the combined trauma significantly increased the levels of inflammatory mediators in BAL fluids of mast cell-competent mice after 3 h, including IL-6, IL-5, MCP-3, eotaxin and MCP-1, compared to the isolated fracture (**Figures 3A** **, C–F**). BAL CXCL-10 levels were also tendentially increased in mast cell-competent mice in the combined trauma group (**Figure 3B**). Other measured inflammatory mediators in BAL fluids are presented in **Table 1**. Notably, pro-inflammatory mediator concentrations in BAL fluids were not significantly reduced in the absence of connective tissue type mast cells (**Figures 3A** **–F**), with the exception of IL-13 and IL-9, the levels of which were significantly lower in mast cell-deficient mice after combined trauma (**Table 1**).

**Figure 4:**
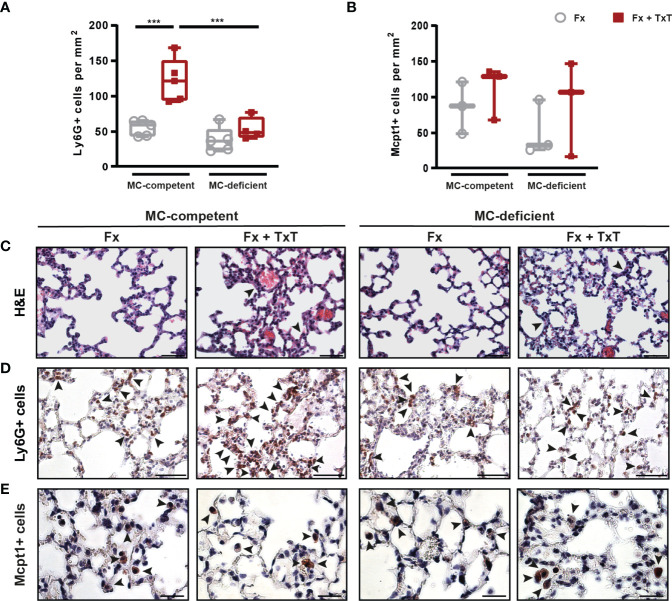
Pulmonary inflammation in mast cell (MC)-competent and MC-deficient mice 3 and 6 h after isolated fracture (Fx) and combined fracture and thoracic trauma (Fx + TxT). **(A)** Number of Ly6G+ cells (neutrophils) in the lung of MC-competent and MC-deficient mice after 3 h. **(B)** Number of Mcpt1+ cells (mucosal mast cells) in the lung of MC-competent and MC-deficient mice after 6 h. **(C)** Representative images of hematoxylin and eosin (H&E) stained lungs of MC-competent and MC-deficient mice. Arrowheads indicate wall thickening. Scale bar = 50 µm. **(D)** Representative images of lungs stained for neutrophils (Ly6G+). Arrowheads indicate positively stained neutrophils. Scale bar = 50 µm. **(E)** Representative images of lungs stained for mucosal mast cells in MC-competent and MC-deficient mice after 6 h. Arrowheads indicate positively stained mast cells (Mcpt-1+). Scale bar = 25µm. Gray boxes represent Fx mice, red boxes represent Fx + TxT mice. Data are shown as box-and-whisker plots (with median and interquartile range) from maximum to minimum, showing all data points. ***p<0.001. The p values were determined using one-way ANOVA with *post hoc* Fisher’s LSD.

Furthermore, the combined trauma caused lung tissue damage and inflammation in mast cell-competent mice, as indicated by the presence of thicker alveolar walls and blood clots 3 h after fracture (**Figure 4C**). This effect was not obviously affected by the absence of connective tissue type mast cells (**Figure 4C**). However, the neutrophil infiltration into the lung was significantly increased only in mast cell-competent mice after combined trauma, but markedly reduced in mast cell-deficient mice (**Figures 4A, D**). Lungs of mast cell-competent and mast cell-deficient mice contained mucosal type mast cells 6 h after trauma (**Figures 4B, E**), but there were no differences visible between the isolated fracture and the fracture and thoracic trauma groups, which was probably influenced by the small sample size and high standard deviations.

### Mast Cell-Deficiency Alters Local Inflammation at the Fracture Site After Trauma

**Figure 5:**
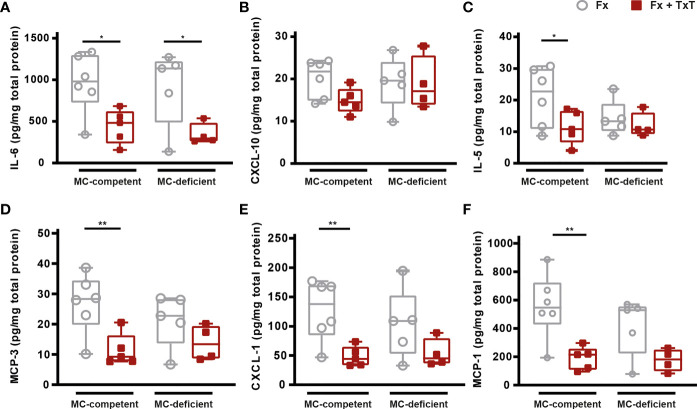
Local inflammation in the fracture hematoma of mast cell (MC)-competent and MC-deficient mice 3 h after isolated fracture (Fx) and combined fracture and thoracic trauma (Fx + TxT). Hematoma **(A)** interleukin-6 (IL-6), **(B)** C-X-C motif chemokine ligand 10 (CXCL-10), **(C)** IL-5, **(D)** monocyte chemoattractant protein-3 (MCP-3), **(E)** CXCL-1, and **(F)** MCP-1 levels. Gray boxes represent Fx mice, red boxes represent Fx + TxT mice. Data are shown as box-and-whisker plots (with median and interquartile range) from maximum to minimum, showing all data points. *p<0.05; **p<0.01. The p values were determined using one-way ANOVA with *post hoc* Fisher’s LSD in the case of normal distribution or with Kruskal-Wallis with *post hoc* Dunn’s test if data were not normally distributed.

To investigate the local inflammation at the fracture site, we determined inflammatory cytokine and chemokine concentrations in the fracture hematoma after 3 h. The combined trauma significantly reduced local levels of inflammatory mediators in the fracture hematoma of mast cell-competent mice, including IL-6, IL-5, CXCL-1, and MCP-1 and -3 (**Figures 5A** **, C–F**). Additionally, hematoma CXCL-10 levels were tendentially reduced in mast cell-competent mice (**Figure 5B**). Notably, these mediators were not reduced in the fracture hematoma of mast cell-deficient mice after combined trauma, with the exception of IL-6, the levels of which were also significantly reduced in mast cell-deficient mice (**Figure 5A**).

### Mast Cell-Deficiency Attenuates Compromised Fracture Healing After Trauma

**Figure 6:**
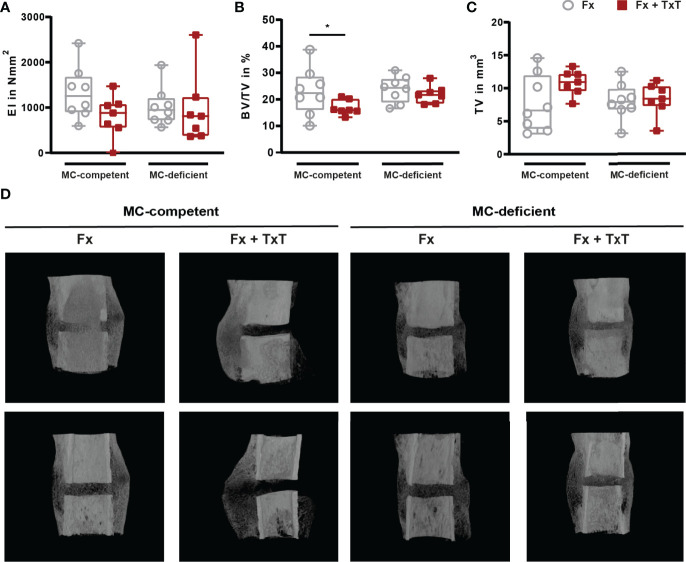
Biomechanical and µCT analyses from fractured femurs of mast cell (MC)-competent and MC-deficient mice 21 d after isolated fracture (Fx) and combined fracture and thoracic trauma (Fx + TxT). **(A)** Bending stiffness (EI) of fractured femurs. **(B)** Bone volume to tissue volume (BV/TV) in the periosteal fracture callus. **(C)** Tissue volume (TV) of the periosteal fracture callus. **(D)** Representative images from µCT three-dimensional reconstructions of the fracture area recorded at similar settings. Gray boxes represent Fx mice, red boxes represent Fx + TxT mice. Data are shown as box-and-whisker plots (with median and interquartile range) from maximum to minimum, showing all data points. *p<0.05. The p values were determined using one-way ANOVA with *post hoc* Fisher’s LSD.

**Figure 7:**
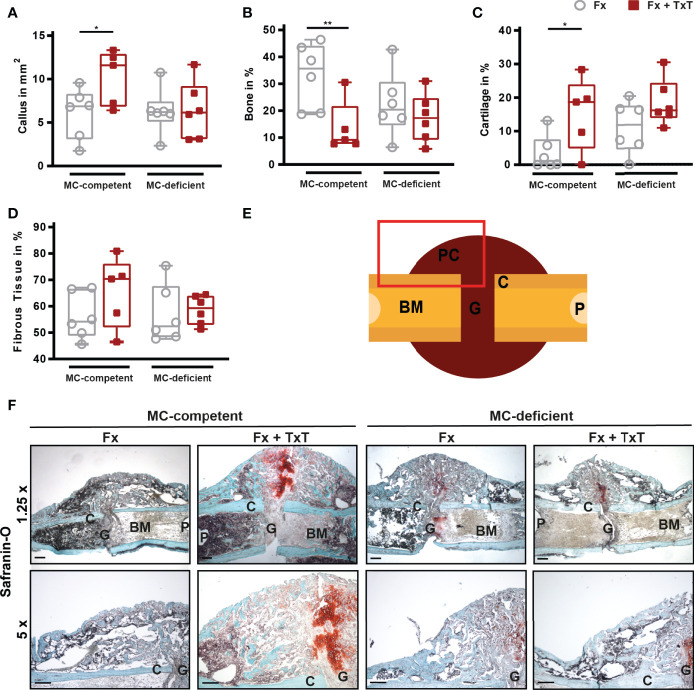
Histomorphometrical analysis from fractured femurs of mast cell (MC)-competent and MC-deficient mice 21 d after isolated fracture (Fx) and combined fracture and thoracic trauma (Fx + TxT). Sections of fractured femurs were stained with Safranin O and **(A)** callus size, **(B)** relative bone area, **(C)** relative cartilage area, and **(D)** relative fibrous tissue area were determined by histomorphometric analysis. **(E)** Schematic illustration of the area (red rectangle) of the fracture callus that was illustrated in F bottom. C = cortex, G = gap, P = pin hole, PC = periosteal callus, BM = bone marrow. **(F)** Representative images from the periosteal fracture callus stained with Safranin O in 1.25 x (top) and 5 x (bottom). Scale bar (top) = 500 µm; scale bar (bottom) = 200 µm. Gray boxes represent Fx mice, red boxes represent Fx + TxT mice. Data are shown as box-and-whisker plots (with median and interquartile range) from maximum to minimum, showing all data points. *p<0.05; **p<0.01. The p values were determined using one-way ANOVA with post-hoc Fisher’s LSD.

On d 21 after fracture, bone repair was assessed by biomechanical testing, µCT, and histomorphometric analyses. In mast cell-competent mice, the combined trauma significantly impaired fracture healing compared to isolated fracture (**Figures 6A** **–D**), as indicated by a slightly reduced bending stiffness of the fractured bones, and a significantly reduced bone volume to tissue volume (BV/TV) of the callus as assessed by µCT analysis (**Figures 6B, D**). Histomorphometric evaluation revealed that the callus size was significantly increased in mast cell-competent mice after combined trauma (**Figures 7A, F**). Furthermore, the combined fracture and thoracic trauma led to a significant reduction of the amount of newly formed bone and a significant increase of the amount of cartilage in the callus of mast cell-competent mice (**Figures 7B, C, F**). The amount of soft tissue was unaffected (**Figures 7D, F**). Notably, mast cell-deficient mice did not display compromised bone healing after combined trauma, because the bone and cartilage contents did not differ compared to mast cell-competent mice with isolated fracture (**Figure 7**), suggesting that mast cells contribute to compromised bone repair after trauma.

**Figure 8:**
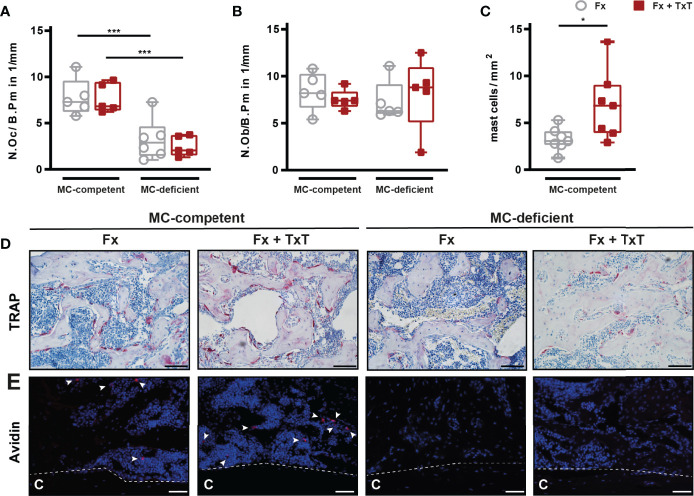
Cellular composition of the fracture callus of mast cell (MC)-competent and MC-deficient mice 21 d after isolated fracture (Fx) and combined fracture and thoracic trauma (Fx + TxT). **(A)** Number of osteoclasts per bone perimeter (N.Oc/B.Pm), **(B)** number of osteoblasts per bone perimeter (N.Ob/B.Pm) and **(C)** number of mast cells per mm^2^. **(D)** Representative images from an area within the periosteal fracture callus stained for TRAP. Scale bar = 100 µm. **(E)** Representative images of mast cell (arrowheads) staining from an area within the periosteal callus. Scale bar = 75 µm. Gray boxes represent Fx mice, red boxes represent Fx + TxT mice, C = cortex. Data are shown as box-and-whisker plots (with median and interquartile range) from maximum to minimum, showing all data points. *p<0.05; ***p<0.001. The p values were determined using one-way ANOVA with post-hoc Fisher’s LSD.

In the fracture callus on d 21, osteoclast numbers were significantly reduced in mast cell-deficient mice with isolated fracture and with the combined trauma compared to the respective mast cell-competent mice (**Figures 8A, D**). By contrast, osteoblast numbers did not differ between mast cell-competent and mast cell-deficient mice of both the isolated fracture and combined trauma groups (**Figure 8B**). Interestingly, in mast cell-competent mice with an additional thoracic trauma, the number of connective tissue type mast cells was significantly increased in the fracture callus on d 21 (**Figures 8C, E**). As expected, in mast cell-deficient mice, there were no connective tissue type mast cells detectable in both groups (**Figures 8C, E**).

## Discussion

The present study investigated the role of mast cells in the pathology of compromised bone fracture healing after severe trauma. Using mast cells-deficient Mcpt5-Cre R-DTA mice that lack connective tissue type mast cells (31), we showed that mast cells contribute to the increased systemic posttraumatic inflammation after severe trauma and a dysregulated early immune response locally at the fracture site, which results in trauma-induced compromised bone repair. These results implicate that mast cells could be a target for new therapeutic strategies to improve bone repair in multiply injured patients.

Mast cells may be the first line of cellular defense after trauma because of their high responsiveness to alarm signals generated after injury such as complement anaphylatoxins or DAMPs, and their release of pre-formed and *de novo* synthesized inflammatory mediators (19). However, nothing is known about their involvement in trauma-induced compromised bone repair, which is frequently observed in multiply injured patients (2). Thereby, we focused on the early inflammatory phase, which is strongly affected by the additional trauma, and on the later repair phase, which serves as a read-out for successful fracture healing, and where mast cells were already shown to play an important role during fracture repair (26, 29). In agreement with previous experimental studies (7, 11, 13, 17, 26, 39–42), the model of combined fracture and thoracic trauma used in the present study induced an increased systemic inflammation after 3 h in mast cell-competent mice as indicated by elevated serum IL-6, CXCL-10, MCP-3, IL-5 and eotaxin levels. IL-6 is one of the key cytokines of posttraumatic inflammation and its level correlates with injury severity and mortality in trauma patients (16, 30, 43). The chemokine CXCL-10 drives leukocyte recruitment and activation (44), whereas IL-5 and eotaxin are key mediators of eosinophil activation (45, 46). MCP-1 is a chemoattractant for monocytes (47), and MCP-3 recruits distinct leukocyte subtypes to injured tissues (48). Moreover, the concomitant thoracic trauma induced pulmonary inflammation, indicated by lung damage, increased inflammatory mediators in the BAL fluids, including IL-6, IL-5, eotaxin, MCP-3, and MCP-1, and increased neutrophil infiltration into the lungs in mast cell-competent mice. These findings are in agreement with previous investigations of trauma models in mice (11, 12, 17, 35) and pigs (14). In the fracture hematoma, the concomitant thoracic trauma significantly reduced the levels of inflammatory mediators in mast cell-competent mice after 3 h. Supporting our data, Horst et al. also found lower IL-6 and IL-8 concentrations locally in the fracture hematoma of pigs after a combined trauma compared to an isolated fracture (42). The deficiency of critical pro-inflammatory mediators during the early inflammatory phase of bone repair was proposed to hamper effective mesenchymal stem cell recruitment and, consequently, bone regeneration (49). Fracture hematoma mediator concentrations are highly dynamic and change rapidly after trauma (13, 42), which is why results might differ at later observation time points. However, as a limitation of our study, we did not include later observation time points during the inflammatory phase. Confirming previous data (11, 12, 17), mast cell-competent mice of the combined trauma group displayed compromised fracture healing at d 21 as indicated by a reduced bone content and increased cartilage residuals in the fracture callus. Interestingly, mast cell numbers were significantly increased in the callus of mast cell-competent mice after combined trauma. This further indicates a direct pathological role of these cells in this context.

Confirming this hypothesis, our study revealed that mast cell-deficient mice displayed a diminished systemic inflammatory response, an altered immune response in the fracture hematoma and uneventful bone repair after combined trauma, indicating that mast cells might contribute to trauma-induced compromised bone repair. Supporting our findings, others previously revealed that mast cell-deficiency increased survival rates after acute sepsis (50). In hemorrhagic shock, mast cell stabilization using cromolyn or doxantrazole, respectively, improved cardiac contractility (25), and the outcome of gut and lung injury in mice (51). Here, we found that mast cell-deficient mice displayed reduced systemic IL-6 and CXCL-10 levels compared to mast cell-competent mice after combined injury. Consequently, mast cells might contribute to posttraumatic inflammation after combined fracture and thoracic trauma by the release of the mentioned factors. Mast cells store considerable amounts of IL-6 and can rapidly *de novo* synthesize IL-6 after activation (52, 53). *In vitro*, mast cells were shown to release IL-6 in response to lipopolysaccharide stimulation (54). In sepsis, mast cells are a critical source for IL-6, because mice with a mast cell-specific IL-6 depletion displayed diminished survival and bacterial clearance in a cecal ligation and puncture model (55). Supporting our findings, systemic cytokine concentrations, including IL-6 levels, were reduced in mast cell-deficient mice after hemorrhagic shock and multiple organ injury was improved (21). Furthermore, mast cell-deficient mice displayed diminished IL-6 levels and improved coronary inflammation in response to stress-induced trauma (56). In addition, systemic inflammatory mediator levels, including IL-6 and CXCL-10, were reduced in mast cell-deficient mice during regular and ovariectomy-induced compromised bone repair, indicating that mast cells contribute to fracture-induced inflammation (26, 29). Mast cell stabilization using cromolyn in mice improved the outcome of ischemic-reperfusion injury associated with reduced blood IL-6 and TNF levels (24). IL-6 is a strong inducer of the hepatic acute-phase response (57), which is initiated after tissue injury or inflammation (58). Interestingly, the posttraumatic liver *Crp* and *Cxcl-1* expression was considerably reduced in mast cell-deficient mice after combined trauma, suggesting that mast cell-released IL-6 might critically regulate the acute-phase response after trauma. Moreover, mast cells are able to produce CXCL-10 (59), which was recently also shown by our group *in vitro* and *in vivo* to be present in the murine fracture callus (29). Supporting this, *in vitro* data showed that mast cells release CXCL-10 upon respiratory virus infection (60), and stimulate airway muscle cells to produce CXCL-10 (61). Furthermore, mast cell numbers correlated with CXCL-10 expression in asthma (62). The IL-5, MCP-3, and eotaxin levels were also significantly reduced in mast cell-deficient mice after combined trauma. It was shown that mast cells produce and release IL-5 in asthma (63), and that mast cell-released IL-5 contributes to intestinal inflammatory disease (64). Both Eotaxin and MCP-3 are produced by mast cells (59), however, nothing is known about the involvement of mast cell-released eotaxin or MCP-3 in other trauma settings. Concluding, our results suggest that mast cell-released inflammatory mediators contribute to trauma-induced systemic inflammation after concomitant thoracic trauma.

In the lungs, MC-deficient mice also displayed an increased pulmonary inflammation in response to the combined trauma, because BAL fluid IL-6, CXCL-10 and MCP-1 levels were elevated. Importantly, the lungs of mice contain both, connective tissue and mucosal mast cells (65, 66). It was previously shown, and is also supported by our findings in the lung, that Mcpt5-Cre R-DTA mice display mucosal mast cells (31), which might explain the increased pulmonary inflammation observed after trauma in both groups. Moreover, other immune cells secrete inflammatory cytokines after lung injury, including tissue-resident macrophages and T cells (67), and could thereby contribute to pulmonary inflammation in our model. However, our study was limited in that we did not investigate further immune cell populations, except neutrophils, in the lungs of our mice. Interestingly, neutrophil infiltration in the lung was reduced in mast cell-deficient mice compared to mast cell competent-mice after thoracic trauma. One possible explanation for this might be that the systemically reduced chemokine concentrations of CXCL-10, eotaxin and MCP-1 in mast cell-deficient mice attenuated neutrophil recruitment from the bone marrow to the inflamed lung (68–70). Supporting this hypothesis, liver *Cxcl-1* expression was significantly reduced in mast cell-deficient mice compared to -competent mice after combined trauma. Alternatively, this might indicate a special role of lung connective tissue type mast cells for neutrophil recruitment into the lung after thoracic trauma. Supporting this, mast cells have been demonstrated to recruit neutrophils, for example, *via* CXCL-1 during tissue inflammation, or IL-1β in urticaria (71, 72). Concluding, while mast cell-deficiency did not abolish trauma-induced lung inflammation, it did influence it.

In contrast to mast cell-competent fracture hematoma, the inflammatory mediators were not reduced in mast cell-deficient mice 3 h after combined fracture and thoracic trauma indicating that mast cells regulate local immune responses at the fracture site after severe injury. Only local IL-6 levels were similarly diminished in mast cell-deficient mice upon the combined trauma, which could be explained by the 3 h observation time point, and the fact that IL-6 is mainly *de novo* synthesized by mast cells, which takes some time (53). Mast cell-specific knockout models could further clarify the role of distinct mast cell-released mediators during uneventful and trauma-induced compromised fracture healing. Furthermore, mast cells are not the single source of IL-6 in the fracture hematoma, because, for example, neutrophils and bone-resident osteoblasts also produce IL-6 locally (12, 36). As a limitation of our study, we did not investigate the immune cell composition in the early fracture hematoma, which would provide additional insights into the interaction of mast cells and other immune cells, including neutrophils, whose numbers are locally increased after severe trauma (11, 17, 40). Regarding this, we previously showed that in regular fracture healing, the neutrophil and macrophage numbers were reduced in the hematoma of mast cell-deficient mice (26). Nevertheless, our present data showed that a balanced local immune response in mast cell-deficient mice resulted in improved bone regeneration 21 days after fracture by enhancing the fracture callus bone content. However, we did not observe any differences in fracture callus osteoblast numbers between mast cell-competent and -deficient mice. Therefore, the improved healing might be a result of an accelerated cartilage-to-bone transition, because the cartilage content was not increased in mast cell-deficient mice with concomitant thoracic trauma. However, limiting our study, we did not investigate an intermediate time point covering the phase of endochondral bone formation, nor a very late time point after fracture, which might have completed the picture of the mechanistic role of mast cells in trauma-induced compromised bone repair. Confirming previous studies, osteoclast numbers did not differ between isolated fracture and the combined trauma groups (35), but osteoclasts were significantly reduced in mast cell-deficient mice of both groups, thereby confirming our previous findings that mast cells play a role in the regulation of osteoclastogenesis during callus remodeling (26, 29). Concluding, our results demonstrated that trauma-induced compromised fracture healing was abolished in mast-cell deficient mice.

In conclusion, the present study demonstrated for the first time that mast cells are involved in the pathomechanisms of compromised bone repair after severe trauma by regulating posttraumatic systemic and local inflammatory reactions. However, additional investigations are needed to elucidate the crosstalk of mast cells and other immune cells, as well as the underlying molecular mechanisms. Nevertheless, our results imply that mast cells could be a target for the development of new therapeutic strategies to improve bone repair in multiply injured patients.

## Data Availability Statement

The original contributions presented in the study are included in the article/supplementary material. Further inquiries can be directed to the corresponding authors.

## Ethics Statement

The animal study was reviewed and approved by Regierungspräsidium Tübingen.

## Author Contributions

DR: conceptualization, investigation, data curation and interpretation, formal analysis, visualization, and writing-original draft. JB: investigation, data curation. KH: investigation, data curation. MV: data curation. MH-L: conceptualization, funding acquisition, methodology, data interpretation, supervision. AD: conceptualization, methodology, data interpretation, supervision, funding aquisition. AI: conceptualization, data interpretation, funding acquisition, writing-original draft, writing-review and editing, supervision. VF: conceptualization, formal analysis, visualization, data curation and interpretation, writing-original draft, writing-review and editing. All authors contributed to the article and approved the submitted version.

## Funding

This research was supported by the German Research Foundation, within the context of the Collaborative Research Center 1149 “Danger Response, Disturbance Factors and Regenerative Potential after Acute Trauma” (Project-ID 251293562, C01 INST 40/491-2), and by grant 361210922/RTG 2408 of the German Research Foundation.

## Conflict of Interest

The authors declare that the research was conducted in the absence of any commercial or financial relationships that could be construed as a potential conflict of interest.

## Publisher’s Note

All claims expressed in this article are solely those of the authors and do not necessarily represent those of their affiliated organizations, or those of the publisher, the editors and the reviewers. Any product that may be evaluated in this article, or claim that may be made by its manufacturer, is not guaranteed or endorsed by the publisher.
